# A metalloproteinase of the disintegrin and metalloproteinases and the ThromboSpondin Motifs 6 as a novel marker for colon cancer: functional experiments

**DOI:** 10.1590/1678-4685-GMB-2019-0266

**Published:** 2020-10-12

**Authors:** Yun-Peng Wang, Yu-Jie Zhao, Xiang-Liang Kong

**Affiliations:** ^1^ The Affiliated Hospital of Shandong University of Traditional Chinese Medicine, Department of Digestive Endoscopy, Jinan, Shandong, P.R. China.

**Keywords:** Colon cancer, A metalloproteinase of the disintegrin and metalloproteinases and the ThromboSpondin Motifs 6, growth, invasion, migration, epithelial–mesenchymal transition

## Abstract

Herein, we aimed to investigate the functions of ADAMTS6 in colon cancer and its potential mechanism. Based on the data acquired from TCGA database, we revealed that ADAMTS6 was highly expressed in colon cancer tissues, and high expression of ADAMTS6 predicted worse prognosis in patients with colon cancer. Moreover, qRT-PCR demonstrated that the levels of ADAMTS6 were higher in colon cancer cell lines (NCI-H508, Caco-2, CW-2 and HCT 116) than that in normal control cell line CCD-18Co. Functional experiments displayed that depletion of ADAMTS6 repressed NCI-H508 cell growth, invasion and migration whilst overexpression of ADAMTS6 facilitated Caco-2 cell growth, invasion and migration. Moreover, ADAMTS6 silencing enhanced the protein expression of E-cadherin and reduced the levels of N-cadherin, Vimentin and Snail in NCI-H508 cells, whereas ADAMTS6 overexpression showed the counter effects in Caco-2 cells. The protein levels of p-AKT and p-p65 were decreased by depletion of ADAMTS6 in NCI-H508 cells, while their levels were enhanced by overexpression of ADAMTS6 in Caco-2 cells. These consequences indicated that the accelerating effect of ADAMTS6 on colon cancer cell growth, migration and invasion might be achieved by modulating EMT and AKT/NF-κB signaling pathway, offering important foundations for colon cancer treatment.

## Introduction

Among the global cancers, the incidence of colon cancer ranks third, and the mortality rate ranks fourth. In 2018, there were 1,096,601 new colon cancer cases and about 551,269 people died of colon cancer (Bray *et al.*, 2018). Hence, the occurrence and metastasis of colon cancers seriously threaten human health. In different colon cancer patients, speed of cancer development and time to metastasis show large differences (Qi and Ding, 2018). The development and progression of colon cancer involves various factors, containing activation of oncogenes and deactivation of tumor suppressor genes (Zhai *et al.*, 2017). Thus, exploring novel molecular markers for colon cancer might contribute to its inchoate diagnosis and therapy.

Recently, studies have discovered that certain changes in the expression of oncogenes or cancer inhibitor genes might result in the occurrence of colon cancer (Lu *et al.*, 2017; Nishikawa *et al.*, 2019). By probing the biological mechanisms of colon cancer, a number of related genes, including oncogenes and tumor suppressor genes have been identified (Xu G *et al.*, 2017). A metalloproteinase of7 the disintegrin and metalloproteinases and the ThromboSpondin Motifs (ADAMTS) family contains 19 members, all of which retain a broadly similar modular structure (Kelwick *et al.*, 2015; Won *et al.*, 2019). According to the study of (Wang *et al.*, 2011), ADAMTS12 expression exerted important impacts on suppressing colorectal cancer development. In esophageal squamous cell carcinoma patients, ADAMTS6 was predicted as a poor prognosis indicator (Liu L *et al.*, 2018). As reported, ADAMTS6 restrained the progression of human breast cancer through ERK pathway (Xie *et al.*, 2016). By bioinformatics analysis of RNA-Seq data, ADAMTS6 was identified as a dysregulated gene for colorectal cancer (Xiao *et al.*, 2015). However, there is few data about the mechanism of the impacts of ADAMTS6 on colon cancer.

In our present study, we found that ADAMTS6 was highly expressed in colon cancer tissues, and high expression of ADAMTS6 was associated with worse overall survival. Furthermore, we illustrated that ADAMTS6 plays a promoting role in the growth, invasion and migration of colon cancer cells, which might be realized by modulating epithelial–mesenchymal transition (EMT) and AKT/NF-κB pathway.

## Material and Methods

### Bioinformatics analysis

The RNA-Seq expression data of 480 colon cancer tissues and 41 normal specimens were acquired from the cancer genome atlas (TCGA) database (https://cancergenome.nih.gov/). According to the median value of ADAMTS6 expression level, patients were fell into high and low expression groups.

### Cell lines and cell culture

Human colon cancer cell lines including NCI-H508, Caco-2, CW-2 and HCT 116, and normal control cell line CCD-18Co were obtained from American Type Culture Collection (ATCC, USA). The cells were maintained in RPMI-1640 medium including 10% fetal bovine serum (FBS) and antibiotics (100 μg/mL streptomycin and 100 U/mL penicillin) under normal conditions.

### Cell transfection

Full length sequences of ADAMTS6 were cloned into pcDNA3.1 vector to over-express ADAMTS6 level in NCI-H508 cells and CCD-18Co cells, and termed pcDNA3.1-ADAMTS6. pcDNA3.1 vector was used as negative control. RNA interference was utilized to silence ADAMTS6 expression in Caco-2 cells. The siRNA sequences for ADAMTS6 were presented in Table 1. The scrambled siRNA was used as a negative control (si-con). pcDNA3.1-ADAMTS6, pcDNA3.1, si-ADAMTS6#1, si-ADAMTS6#2 and si-con were synthesized by Shanghai GenePharma Co., Ltd. (Shanghai, China) and were transfected into cells using the Lipofectamine2000 transfection kit (Invitrogen, Shanghai, China).

**Table 1:** The sequences used in cell transfection.

Name	Sequences
si-con	5’-CGAACUCACUGGUCUGACC-3’
si- ADAMTS6#1	5’-CTTAGTTAAAGGTGTCC-3’
si- ADAMTS6#2	5’-AGTGCAAAGATGTGAAT-3’

### qRT-PCR

After 48 h transfection, total RNA was extracted from cultured cells utilizing Trizol (Invitrogen) and reverse transcribed using a reverse transcriptase kit (Takara, Dalian, China) following the protocol. Employing ABI7300 System (USA) with SYBR Green, qPCR was executed to quantify the expressions of ADAMTS6 along with GAPDH as an internal control. The expression of ADAMTS6 was calculated by 2^-^ΔΔct method. Table 2 lists the primers used for qPCR.

**Table 2:** The primers utilized in qRT-PCR.

Name	Sequences
ADAMTS6 forward	5’-GTGATCCTGACAGTAAGCCACC-3’
ADAMTS6 reverse	5’-CCACCATCACAAGTCTTGCTGC-3’
GAPDH forward	5’-TGTGTCCGTCGTGGATCTGA-3’
GAPDH reverse	5’-CCTGCTTCACCACCTTCTTGA-3’

### Western blotting

The transfected cells were lysed utilizing RIPA lysis buffer. Twenty μg samples were heated at 95 °C for 5 min, and separated by 10% SDS-PAGE. Then the proteins were transferred onto a PVDF membrane. After being blocked with 5% skim milk for one hour, the membrane was incubated with primary antibodies ADAMTS6 (1:1000, Elabscience Biotechnology Co., Ltd., Wuhan, China), E-cadherin (1:1000, #3195, Cell Signaling Technology, Inc.), N-cadherin (1:1000, #13116, CST), Vimentin (1:1000, #5741, CST), Snail (1:1000, #3879, CST), GAPDH (1:5000, #5174, CST) at 4 °C overnight and the secondary antibodies (1:5,000, #7074, CST) at room temperature for one hour. Subsequently, the proteins were visualized utilizing an ECL system. An ImageJ software (Bio-Rad, Hercules, CA) was utilized to quantify the protein expression. GAPDH was utilized as a control. The blotting was repeated in three times, independently.

### Cell counting

A CCK-8 Kit (YEASEN, Shanghai, China) was applied to evaluate cell proliferation. After 48 h transfection, 1 × 10^3^ cells were incubated in 96-well plates under standard conditions. Then, each well was added with 10 μL of CCK-8 solution at 24, 48, 72 h time points. After the cells were incubated for another 1.5 hours, the OD_450_ was detected utilizing a microplate reader (Tokyo, Japan).

### Colony formation assay

Transfected colon cancer cells were plated in 6-well plates at a density of 400 cells per well, then cultured in completely medium supplemented with 10% FBS with 5% CO_2_ at 37 °C for 14 days, and refreshed every three days. Then the cells were washed three times with PBS and then stained with 0.1% Crystal Violet solution for 15 min. Finally, the number of colonies formed in the plates was counted.

### Cell migration and invasion assay

The tumor cell migration and invasion capacity was measured by transwell assay. The chambers were coated with (for invasive ability determination) or without (for the migration test) matrigel (BD Biosciences, USA). Cell suspension was prepared with serum-free medium after 24 h transfection and 1×10^6^ cells were added to the upper chamber. Then, 500 μL of completely medium was filled into the lower chamber. Then the cells were cultured for 48 h under normal condition. The migrated and invaded cells were fixed with 4% paraformaldehyde for 30 min and then stained with 0.1% Crystal Violet for 30 min. Finally, cell number was counted under a microscope (Nikon, Japan) from 5 random fields.

### Assessment of wound healing

Wound-healing assay was executed to further test cell migration. Transfected colon cancer cells were seeded into 24-well plates at 5 × 10^5^ cells/mL. After the cells being famished for 24 h, the scratches were created by utilizing a sterile 200 μL tip. The wound closure was captured at 0 h and 24 h under an optical microscope.

### Statistical analysis

To assess the prognostic value of ADAMTS6 in colon cancer, the Kaplan-Meier method was utilized and the statistical significance was assessed by the log-rank test. To investigate whether ADAMTS6 can serve as an independent predictor of colon cancer prognosis, cox regression analysis was carried out.

The statistical analysis was done utilizing SPSS 22.0 statistical software and GraphPad Prism 7.0 (GraphPad Software, USA). The differences of the results between 2 groups were analyzed by Student's *t*-test. To compare more than 2 groups, one-way analysis of variance (ANOVA) with Bonferroni post hoc test was utilized. All data are represented as Mean ± Standard Deviation (SD). Differences were considered statistically significant when P <0.05.

## Results

### High expression of ADAMTS6 predicts poor prognosis in colon cancer patients

**Figure 1:**
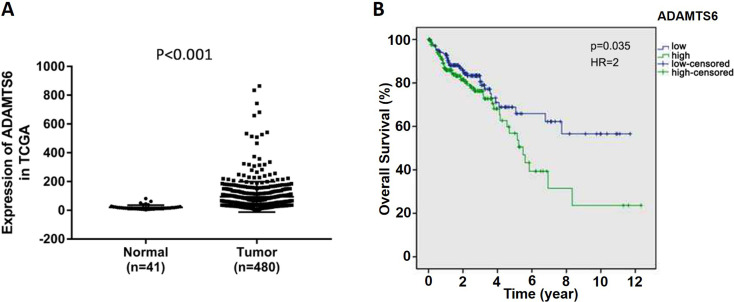
High expression of ADAMTS6 predicts poor prognosis in colon cancer. (A) The mRNA level of ADAMTS6 in colon cancer tissue and normal samples. (B) Kaplan-Meier was utilized to analyze the overall survival of ADAMTS6 in colon cancer patients. ADAMTS6 high expression was concerned with a worse prognosis in colon cancer relative to the ADAMTS6 low expression group.

To determine the ADAMTS6 level in colon cancer, the expression differences of ADAMTS6 between colon cancer tissues and normal samples were investigated utilizing public RNA-Seq data from TCGA database. The outcomes displayed that the expression of ADAMTS6 was higher in colon cancer tissues compared to that in normal samples (Figure 1A,P <0.001). The association between ADAMTS6 expression and the overall survival of patients with colon cancer was explored utilizing Kaplan-Meier Plotter. The consequences demonstrated that the overall survival of patients with high expression of ADAMTS6 was shorter than that with low expression of ADAMTS6 (Figure 1B,P = 0.035, HR=2). The univariate analysis revealed that the analyzed variables (ADAMTS6 expression, Pathologic-stage and Pathologic-TMN) were strongly related to the overall survival time of colon cancer patients. Further multivariate analysis uncovered that Pathologic-T (P = 0.028) and Pathologic-M (P <0.001) were promising independent prognostic factors of colon cancer (Table 3). However, ADAMTS6 could not be used as an independent prognostic factor. Taken together, these consequences suggested the involvement of ADAMTS6 in colon cancer progression and indicated that high expression of ADAMTS6 might predict worse prognosis in colon cancer patients.

**Table 3:** Cox proportional hazards model analysis of clinic pathologic features for overall survival of ADAMTS6 in colon cancer patients.

Variables	Univariate analysis			Multivariate analysis		
	HR	95%CI	P value	HR	95%CI	P value
Expression						
Low	1	1				
High	1.522	1.029-2.341	0.036*	1.239	0.791-1.943	0.35
Pathologic-Stage						
I+II	1	1				
III+IV	2.805	1.812-4.343	<0.001*	2.322	0.692-7.793	0.173
Pathologic-T						
T1+T2	1	1				
T3+T4	3.697	1.498-9.122	0.005*	9.293	1.268-68.133	0.028*
Pathologic-M						
M0	1	1				
M1	4.839	3.037-7.710	<0.001*	2.973	1.669-5.296	<0.001*
Pathologic-N						
N0	1	1				
N1+N2	2.529	1.667-3.835	<0.001*	0.625	0.216-1.810	0.386
Age						
<60	1					
[#GTEQ#]60	1.356	0.834-2.205	0.219			
Gender						
Female	1					
Male	1.209	0.803-1.821	0.363			

* P<0.05, HR: hazard ratio.

### Overexpression and knockdown of ADAMTS6 in colon cancer cell lines

**Figure 2:**
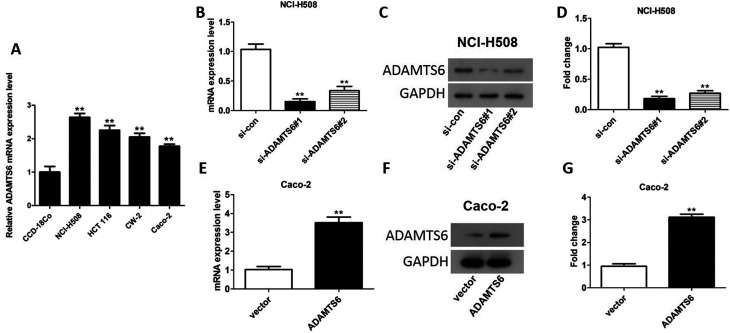
The expression of ADAMTS6 in colon cancer cell lines. (A) The expression of ADAMTS6 in CCD-18Co, NCI-H508, Caco-2, CW-2 and HCT 116 cell lines. **P <0.01 vs. CCD-18Co. (B) The mRNA expression of ADAMTS6 in NCI-H508 cells. (C, D) The protein levels of ADAMTS6 in NCI-H508 cells. (E) The mRNA levels of ADAMTS6 in Caco-2 cells. (F, G) The protein levels of ADAMTS6 in Caco-2 cells. **P <0.01 vs. si-control or vector group.

We then studied the expression of ADAMTS6 in 4 different colon cancer cell lines NCI-H508, Caco-2, CW-2 and HCT 116 and a normal human colon cell line CCD-18Co by qRT-PCR. Compared to CCD-18Co cells, an obviously increase of ADAMTS6 expression was found in these 4 colon cancer cell lines. Furthermore, ADAMTS6 mRNA expression was relatively higher expressed in NCI-H508 cells and was lower expressed in Caco-2 cells compared to other tested colon cancer cells (Figure 2A). Therefore, in the following assays, detection of ADAMTS6 knockdown effect was executed in NCI-H508 cell line and detection of the influences of ADAMTS6 overexpression was carried out in Caco-2 cell line. As illustrated in Figure 2B,D, si-ADAMTS6#1 and si-ADAMTS6#2 lessened the expression of ADAMTS6 both at mRNA and protein levels in NCI-H508 cells. Besides, the knockdown efficiency of si-ADAMTS6#1 was higher than si-ADAMTS6#2, thus si-ADAMTS6#1 was utilized in the follow-up experiments. Furthermore, as shown in Figure 2 E,G, pcDNA3.1-ADAMTS6 could elevate the expression of ADAMTS6 both at mRNA and protein levels in Caco-2 cells.

### The effect of ADAMTS6 on the proliferation of colon cancer cells

**Figure 3:**
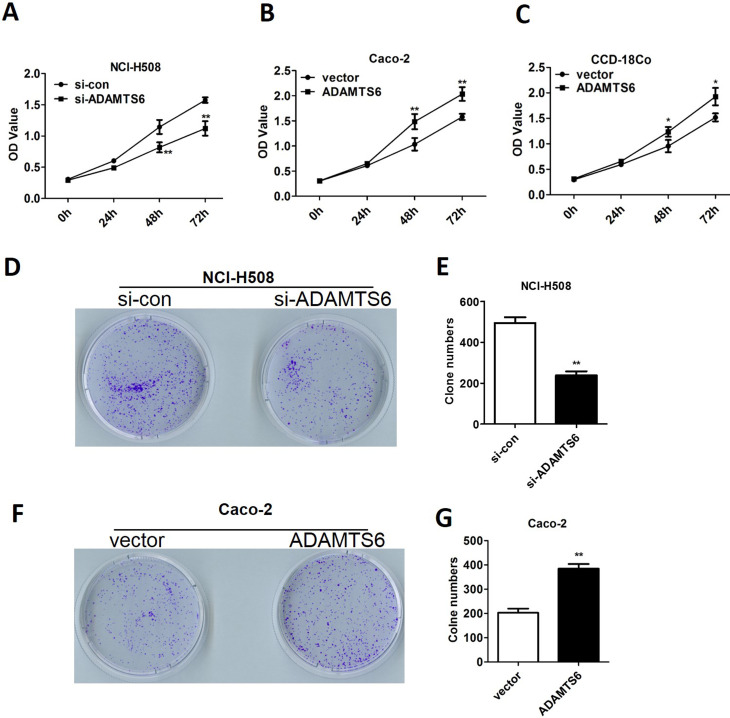
The effect of ADAMTS6 on the proliferation of NCI-H508, Caco-2 and CCD-18Co cells. (A) CCK8 analysis indicated that silencing of ADAMTS6 restrained cell proliferation in NCI-H508 cells. **P <0.01 compared with si-con group. (B-C) CCK8 analysis indicated that the overexpression of ADAMTS6 facilitated cell proliferation in Caco-2 cells (B) and CCD-18Co cells (C). ** P <0.01 compared with vector group. (D-E) The analysis of colony formation rates in si-con and si-ADAMTS6 groups. **P <0.01 compared with si-con group. (F-G) The analysis of colony formation rates in vector and pcDNA3.1-ADAMTS6 groups. ** P <0.01 compared to vector group.

A CCK-8 assay was then performed to explore the effect of ADAMTS6 on the viability of normal colon cells and colon cancer cells. As shown in Figure 3A, the outcomes revealed that the OD_450_ value of NCI-H508 cells reduced in si-ADAMTS6 group relative to that in si-control group, proving that ADAMTS6 knockdown reduced NCI-H508 cell proliferation (P <0.01). Overexpression of ADAMTS6 accelerated Caco-2 cell proliferation after being cultured for 48 h and 72 h (P <0.01), but there was no significant effect at 24 h (Figure 3B). Moreover, the data showed that the OD value of CCD-18Co cells was increased in the ADAMTS6 group than that in the vector group (P <0.05, Figure 3C). These data suggested that ADAMTS6 could promote the proliferation of both colon cancer cells and normal colon cells, and the promoting effect is stronger in colon cancer cells. As the purpose of our study was to investigate the influence of ADAMTS6 on the colon cancer, therefore the following experiments were only performed using colon cancer cell lines.

Colony formation was further assayed to determine the effect of ADAMTS6 on colon cancer cell proliferation. Consistent with the results of the CCK-8 assay, the colony formation ability of NCI-H508 cells was restrained by silencing of ADAMTS6 when compared with si-control group (P <0.01, Figure 3D,E). Besides, the colony formation ability of Caco-2 cells was boosted after overexpression of ADAMTS6 when compared with vector group (P <0.01, Figure 3F,G). All the above findings indicated that ADAMTS6 depletion had a repressive effect and ADAMTS6 overexpression had a stimulative influence on colon cancer cell growth.

### Ablation of ADAMTS6 suppresses cell invasion and migration in NCI-H508 cells while overexpression of ADAMTS6 augments cell invasion and migration in Caco-2 cells

**Figure 4:**
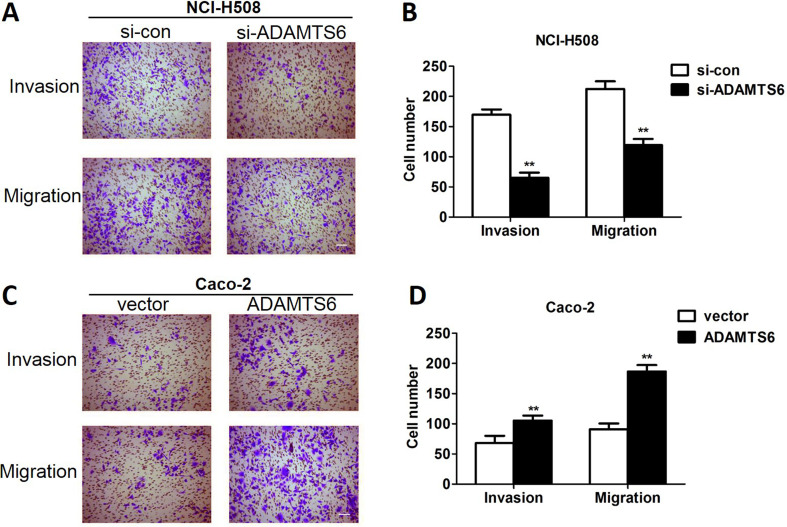
Depletion of ADAMTS6 suppressed the invasion and migration of NCI-H508 cells and ADAMTS6 overexpression accelerated Caco-2 cell invasion and migration. (A) Bright field images of transwell assay in NCI-H508 cells were acquired by an inverted microscope (bar = 100 μm). (B) Quantification of (A). (C) Bright field images of transwell assay in Caco-2 cells were acquired by an inverted microscope (bar = 100 μm). (D) Quantification of (C). **P <0.01 vs. si-control or vector group.

**Figure 5:**
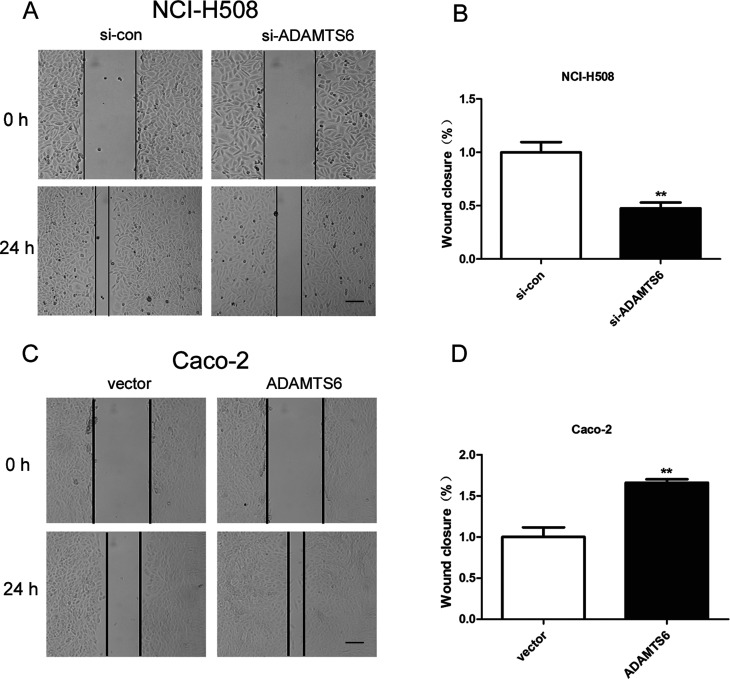
ADAMTS6 facilitated colon cancer cell migration in wound-healing assay. (A) Cell culture images of the wound healing assay in each group were acquired by microscopy at 0 and 24 h after scratching (bar = 200 μm). (B) Wound closure analysis displayed the impact of ADAMTS6 depletion on NCI-H508 cell migration. **P <0.01 vs. si-control group. (C) Cell culture images of the wound healing assay in each group were gained by microscopy at 0 and 24 h after scratching (bar = 200 μm). (D) Wound closure analysis showed the influence of ADAMTS6 overexpression on Caco-2 cell migration. **P <0.01 vs. vector group.

The impact of ADAMTS6 on the invasion and migration of NCI-H508 and Caco-2 cells were investigated utilizing transwell assays. ADAMTS6 deficiency significantly reduced the number of crystal violet-stained NCI-H508 cells in the invasion and migration assays (P <0.01, Figure 4A,B). On the contrary, overexpression of ADAMTS6 increased the number of crystal violet-stained Caco-2 cells in transwell invasion and migration assays (P <0.01, Figure 4C,D). Subsequently, we conducted wound-healing assays to further examine cell migration in colon cancer cells and the results indicated that ADAMTS6 depletion significantly decreased NCI-H508 cell migration (P <0.01, Figure 5A,B), whilst ADAMTS6 overexpression fortified the migratory ability of Caco-2 cells (P <0.01, Figure 5C,D). This indicated that silencing of ADAMTS6 repressed cell invasion and migration in NCI-H508 cells and overexpression of ADAMTS6 increased Caco-2 cell invasion and migration.

### ADAMTS6 can regulate EMT in NCI-H508 and Caco-2 cells

**Figure 6:**
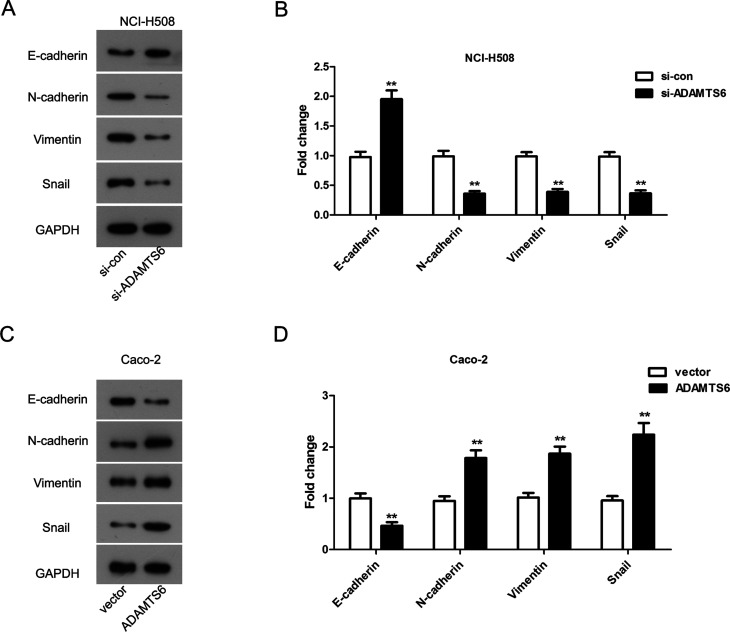
The effect of ADAMTS6 on EMT-related markers was detected by western blotting. (A and B) The expression of E-cadherin, N-cadherin, Vimentin and Snail in NCI-H508 cells. ** P <0.01 vs. si-control group. (C and D) The expression of E-cadherin, N-cadherin, Vimentin and Snail in Caco-2 cells. ** P <0.01 vs. vector group.

EMT is critical for cell motility of colon cancer and E-cadherin, N-cadherin, Vimentin and Snail are the markers of EMT (Zhou *et al.*, 2017; Sun *et al.*, 2019). To gain a deeper understanding on the mechanism of how ADAMTS6 affects cell growth and motility of colon cancer cells, we tested the protein levels of E-cadherin, N-cadherin, Vimentin and Snail by western blotting. As shown in Figure 6A,B, the protein level of E-cadherin was obviously up-regulated by silencing of ADAMTS6 in NCI-H508 cells (P <0.01). While knockdown of ADAMTS6 visibly lessened the protein expression levels of N-cadherin, Vimentin and Snail (P <0.01, Figure 6A,B) in NCI-H508 cells. Moreover, overexpression of ADAMTS6 apparently reduced the protein expression level of E-cadherin in Caco-2 cells (P <0.01, Figure 6C-D). Nevertheless, the protein levels of N-cadherin, Vimentin and Snail were remarkably improved by overexpression of ADAMTS6 (P <0.01, Figure 6C,D) in Caco-2 cells. Together, these results provided important insights into that ADAMTS6 regulated colon cancer cell motility partially through EMT.

### AKT and NF-κB activation facilitates the impact of ADAMTS6 in colon cancer cells

**Figure 7:**
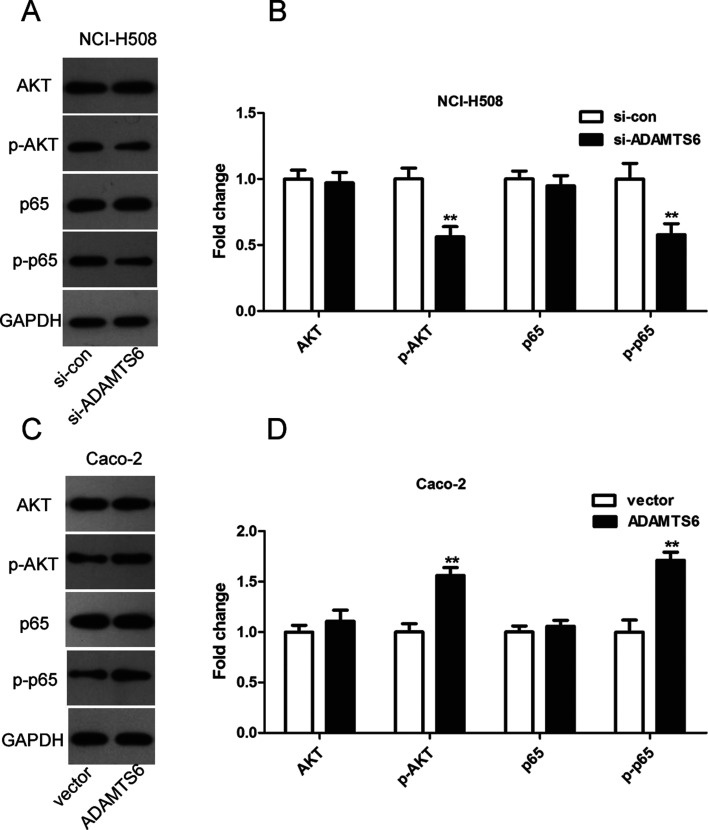
The effect of ADAMTS6 on the activation of AKT and NF-κB in colon cancer cells was detected by western blotting. (A and B) The expression of AKT, p-AKT, p65 and p-p65 in NCI-H508 cells. ** P <0.01 vs. si-control group. (C and D) The expression of AKT, p-AKT, p65 and p-p65 in Caco-2 cells. ** P <0.01 vs. vector group.

We further analyzed the influences on AKT/NF-κB pathway in colon cancer cells to probe the molecular mechanisms responsible for ADAMTS6-mediated EMT promotion. As presented in Figure 7A,B, transfection of si-ADAMTS6 into NCI-H508 cells resulted in low protein levels of p-AKT and p-p65 compared to si-con group (P <0.01). Whereas, overexpression of ADAMTS6 increased the protein levels of p-AKT and p-p65 in Caco-2 cells (P <0.01, Figure 7C,D). These data suggested that AKT and NF-κB activations are vital for the facilitating effects of ADAMTS6 during EMT progression in colon cancer cells.

## Discussion

Based on reported studies, we are the first to exhibit the roles of ADAMTS6 in colon cancer. To be specific, we disclosed that ADAMTS6 was highly expressed in colon cancer tissues and high expression of ADAMTS6 was related to worse prognosis in colon cancer patients. Furthermore, ADAMTS6 depletion dramatically repressed the growth, invasion and migration of NCI-H508 cells, while ADAMTS6 overexpression significantly facilitated Caco-2 cell growth, invasion and migration, which all might through activating EMT and AKT/NF-κB pathway. Therefore, ADAMTS6 might be regarded as a novel promising target for the treatment of colon cancer.

Members of the ADAMTS family display different expression levels in cancer tissues. ADAMTS-1, −3, −5, −8 and −18 were down-regulated in cancer tissues whilst ADAMTS-4, −6, −10 and −14 were overexpressed in cancer tissues (Wagstaff *et al.*, 2011). It has been demonstrated that ADAMTS18, as a novel cancer regulator, was vital to colorectal cancer pathology (Lu *et al.*, 2017). There is one study indicated that ADAMTS6 repressed breast cancer development through modulating the ERK pathway (Xie *et al.*, 2016). Subsequently, ADAMTS6 expression was shown to be distinctly higher in esophageal squamous cell carcinoma tissues (Liu L *et al.*, 2018). Our outcomes manifested that ADAMTS6 overexpression was concerned with worse prognosis of colon cancer patients. Until now, it has not been reported whether ADAMTS6 has a role in colon cancer proliferation, migration and the underlying mechanisms. In the present study, we first revealed that ADAMTS6 deficiency inhibited colon cancer cell proliferation, invasion and migration, whilst high expression of ADAMTS6 accelerated cell growth, invasion and migration in colon cancer cells.

In this study, we also discovered that the impacts of ADAMTS6 on colon cancer cell growth, invasion and migration was potentially modulated by EMT and the AKT/NF-κB pathway. EMT is an important process in cancer that affects the pivotal process by transforming epithelial cells into cells with mesenchymal properties (Zhou *et al.*, 2017). When EMT occurs, E-cadherin (a pivotal cell-to-cell adhesion molecule) expression is reduced, whilst N-cadherin (concerned with a cadherin switching process), Vimentin (a key effect in cell migration) and Snail (an EMT-related transcription factor) are up-regulated (Cappellesso *et al.*, 2015; Seton-Rogers, 2016; Zhou *et al.*, 2017). As reported, EMT plays a vital role in driving colon cancer invasion and metastasis (Lee *et al.*, 2016; Zhu *et al.*, 2016). AKT signaling has a vital effect in EMT, ulteriorly resulting in cancer invasion and migration (Larue and Bellacosa, 2005). Meanwhile, AKT can release NF-κB to come in the nucleus and up-regulate Snail expression to facilitate EMT (Saegusa *et al.*, 2009; Liu W *et al.*, 2018). NF-κB played vital roles in tumor cell proliferation, differentiation, invasion and metastasis (Xia *et al.*, 2014). There are five subunits (Rel, RelB, p50, p52 and p65) in NF-κB family (Espín-Palazón and Traver, 2016). Among these subunits, p65 can modulate the activity of transcription and accelerate the connection of p50 and DNA by coordinating the homologous domains of Rel (Li and Zhao, 2018). ADAMTS9 has been identified as a cancer-suppressor gene in gastric cancer and nasopharyngeal carcinoma (Lung *et al.*, 2012; Du *et al.*, 2013). Of note, it has been indicated that the ADAMTS9 might execute its suppressive function in gastric cancer by repressing AKT/mTOR pathway (Du *et al.*, 2013). Furthermore, it has been revealed that ADAMTS18 could suppress EMT and deregulate AKT and NF-κB signaling in breast tumorigenesis (Xu H *et al.*, 2017). Hence, we inferred that ADAMTS6, as another member of the same subgroup, might affect EMT and AKT/NF-κB in tumor as well. As expected, our present research demonstrated that ADAMTS6 ablation greatly enhanced E-cadherin expression level, but reduced N-cadherin, Vimentin, Snail, p-AKT and p-p65 expression levels in NCI-H508 cells. Moreover, we found that overexpression of ADAMTS6 suppressed E-cadherin expression, but enhanced N-cadherin, Vimentin, Snail, p-AKT and p-p65 expression levels in Caco-2 cells. These outcomes offer a possible mechanism indicating that ADAMTS6 might act as an important contributor on EMT progression and AKT/NF-κB pathway, therefore accelerating colon cancer cell growth, migration and invasion.

Taken together, we argued that ADAMTS6 was highly expressed in colon cancer tissues and its high expression was associated with worse overall survival in colon cancer patients for the first time. Additionally, ablation of ADAMTS6 markedly restrained cell growth, invasion and migration in NCI-H508 cells, while ADAMTS6 overexpression prominently facilitated cell growth, invasion and migration in Caco-2 cells, which all might be modulated by EMT and the AKT/NF-κB pathway. Our observations will offer novel insight into the modulation of ADAMTS6 on the pathogenesis of colon cancer and provide a potential biomarker for colon cancer patients. However, more *in-vivo* studies are needed to ulteriorly verify our findings.
